# Ammonia Oxidation by the Arctic Terrestrial Thaumarchaeote *Candidatus* Nitrosocosmicus arcticus Is Stimulated by Increasing Temperatures

**DOI:** 10.3389/fmicb.2019.01571

**Published:** 2019-07-17

**Authors:** Ricardo J. Eloy Alves, Melina Kerou, Anna Zappe, Romana Bittner, Sophie S. Abby, Heiko A. Schmidt, Kevin Pfeifer, Christa Schleper

**Affiliations:** ^1^Archaea Biology and Ecogenomics Division, Department of Ecogenomics and Systems Biology, University of Vienna, Vienna, Austria; ^2^Max F. Perutz Laboratories, Center for Integrative Bioinformatics Vienna, Medical University of Vienna, University of Vienna, Vienna, Austria; ^3^Institute for Synthetic Bioarchitectures, University of Natural Resources and Life Sciences, Vienna, Austria

**Keywords:** ammonia oxidation, archaea, thaumarchaeota, nitrification, arctic ecosystems, soil microbiology

## Abstract

Climate change is causing arctic regions to warm disproportionally faster than those at lower latitudes, leading to alterations in carbon and nitrogen cycling, and potentially higher greenhouse gas emissions. It is thus increasingly important to better characterize the microorganisms driving arctic biogeochemical processes and their potential responses to changing conditions. Here, we describe a novel thaumarchaeon enriched from an arctic soil, *Candidatus* Nitrosocosmicus arcticus strain Kfb, which has been maintained for seven years in stable laboratory enrichment cultures as an aerobic ammonia oxidizer, with ammonium or urea as substrates. Genomic analyses show that this organism harbors all genes involved in ammonia oxidation and in carbon fixation via the 3-hydroxypropionate/4-hydroxybutyrate cycle, characteristic of all AOA, as well as the capability for urea utilization and potentially also for heterotrophic metabolism, similar to other AOA. *Ca*. N. arcticus oxidizes ammonia optimally between 20 and 28°C, well above average temperatures in its native high arctic environment (−13–4°C). Ammonia oxidation rates were nevertheless much lower than those of most cultivated mesophilic AOA (20–45°C). Intriguingly, we repeatedly observed apparent faster growth rates (based on marker gene counts) at lower temperatures (4–8°C) but without detectable nitrite production. Together with potential metabolisms predicted from its genome content, these observations indicate that *Ca*. N. arcticus is not a strict chemolithotrophic ammonia oxidizer and add to cumulating evidence for a greater metabolic and physiological versatility of AOA. The physiology of *Ca*. N. arcticus suggests that increasing temperatures might drastically affect nitrification in arctic soils by stimulating archaeal ammonia oxidation.

## Introduction

High latitude ecosystems are warming faster than those at lower latitudes and soils in these regions are expected to exhibit higher respiration rates and overall turnover of carbon and nitrogen under rising temperatures (IPCC, 2013). Arctic and boreal soils store particularly large amounts of organic matter, and considerable carbon losses as carbon dioxide (CO_2_) emissions have been observed in these ecosystems under a temperature increase of just 1°C (Crowther et al., 2016). Such climate feedbacks are expected to increase greatly due to permafrost thawing, higher rates of soil organic matter decomposition and consequent higher nutrient bioavailability (particularly of nitrogen), eventually leading to higher greenhouse gas emissions, including CO_2_, methane, and nitrous oxide (N_2_O) (e.g., Dorrepaal et al., 2009; Wild et al., 2014; Capek et al., 2015; Voigt et al., 2017). It is therefore of major importance for future climate projections to better characterize microorganisms that drive biogeochemical processes underlying nutrient and greenhouse gas fluxes in arctic soil ecosystems, such as nitrification, the conversion of ammonia via nitrite to nitrate. Ammonia-oxidizing archaea (AOA) are widespread and abundant in arctic soils, where they generally outnumber ammonia-oxidizing bacteria (AOB) (Siciliano et al., 2009; Lamb et al., 2011; Banerjee and Siciliano, 2012; Daebeler et al., 2012; Alves et al., 2013) and are likely the main drivers of nitrification, at least in some soils (Alves et al., 2013). Together, these observations indicate that AOA play a central role in nitrogen cycling in these nitrogen-limited and particularly sensitive ecosystems. AOA represent the taxonomic class *Nitrososphaeria* of the phylum Thaumarchaeota and are globally distributed in aquatic and terrestrial ecosystems (Brochier-Armanet et al., 2008; Stieglmeier et al., 2014a; Alves et al., 2018). AOA are typically the dominant and often the only detectable ammonia oxidizers in extreme and oligotrophic environments (Hatzenpichler, 2012; Stahl and de la Torre, 2012; Alves et al., 2018), although they are also abundant in highly organic and nutrient-rich environments, such as fertilized soils, peatlands and wastewater treatment systems (Stopnisek et al., 2010; Limpiyakorn et al., 2013; Sauder et al., 2017; Alves et al., 2018).

Although all cultivated AOA strains grow chemolithoautotrophically through oxidation of ammonia, several studies have suggested that growth of at least some AOA predominant in soils and freshwater might not depend solely, or primarily, on ammonia oxidation (see for example Jia and Conrad, 2009; Xu et al., 2012; Alves et al., 2018; Nelkner et al., 2019 for overview). Incorporation of carbon from organic substrates into AOA cells has also been reported in ocean waters (Ouverney and Fuhrman, 2000; Ingalls et al., 2006; Hansman et al., 2009), and other studies were unable to detect autotrophic carbon assimilation in populations actively growing in wastewater treatment plants (Mussmann et al., 2011; Sauder et al., 2017). Additionally, a recent meta-analysis revealed that the two clades of AOA most frequently detected in terrestrial environments lack cultivated representatives and have never been detected in active autotrophic nitrifying communities based on ^13^CO_2_ stable isotope probing (Alves et al., 2018). Together, these observations strongly indicate that at least some lineages of AOA may rely on alternative or complementary and yet unknown carbon assimilation pathways, and that their metabolic spectrum is broader than currently assumed.

Here, we describe the cultivation and genome properties of an ammonia-oxidizing thaumarchaeote enriched from a mineral arctic soil from Svalbard, *Candidatus* Nitrosocosmicus arcticus strain Kfb, affiliated with the candidate genus Nitrosocosmicus, which represent the recently defined *amoA* gene clade NS-ζ (Alves et al., 2018), formerly referred to as *Nitrososphaera*-sister clade (Pester et al., 2012). Three ammonia-oxidizing *Ca*. Nitrosocosmicus strains have been enriched earlier from different environments, namely *Ca*. Nitrosocosmicus franklandus C13 from an agricultural soil (Lehtovirta-Morley et al., 2016), *Ca*. Nitrosocosmicus oleophilus MY3 (Jung et al., 2016) from hydrocarbon-contaminated terrestrial sediments and *Ca*. Nitrosocosmicus exaquare G61 from a wastewater treatment system (Sauder et al., 2017). *Ca*. N. arcticus Kfb is an ammonia oxidizer that grows optimally in enrichment cultures at 20–28°C with ammonium (N${\text{H}}_{4}^{+}$) or urea as substrates, although at extremely low rates. Intriguingly, indications for faster growth rates were repeatedly observed at 4°C (and to a lesser extent at 8°C) without detectable ammonia oxidation, although we could not identify alternative or complementary substrates, or the metabolism supporting this growth behavior.

## Materials and Methods

### Enrichment and Cultivation of AOA

AOA were enriched and routinely cultivated in 30 mL polystyrene vials containing 16 mL Fresh Water Medium (FWM) (Tourna et al., 2011) and 4 mL inoculum [20% (v/v)]. FWM contained NaCl (1 g L^−1^), MgCl_2_·6H_2_O (0.4 g L^−1^), CaCl_2_·2H_2_O (0.1 g L^−1^), KH_2_PO_4_ (0.2 g L^−1^) and KCl (0.5 g L^−1^), FeNaEDTA solution (7.5 μM), 1 mL non-chelated trace element mixture, and 1 mL vitamin solution (Tourna et al., 2011). All cultures were routinely supplemented with NaHCO_3_ (2 mM), streptomycin (100 μg mL^−1^), and 0.5 mM NH_4_Cl or 1 mM urea. Addition of the antibiotics kanamycin, carbenicillin and ampicillin (100 μg mL^−1^) was also individually tested (each in addition to streptomycin), but since they frequently led to inhibition of ammonia oxidation, further cultures were only supplemented with streptomycin. Enrichments cultures were routinely inspected for bacterial contaminants using phase contrast microscopy and end-point PCR targeting bacterial 16S rRNA genes. Despite continuous efforts, we could not completely eliminate the bacterial contaminants. All solutions were prepared with milli-Q water and autoclaved, or filter-sterilized in the case of heat-sensitive compounds (i.e., vitamins and FeNaEDTA). The final pH of the default medium ranged between 7.0 and 7.5; initial attempts to buffer the medium using HEPES lead to inhibition of ammonia oxidation, and thus all subsequent cultivation was performed in unbuffered medium. All enrichment cultures were incubated in the dark, and ammonia-oxidizing cultures were routinely incubated at 20°C, the temperature at which ammonia oxidation was most stable. The default medium and incubation conditions were used for cultivation experiments with different supplements and NH_4_Cl/urea concentrations, or under different temperatures or pH, unless otherwise stated. Concentrations of N${\text{O}}_{2}^{-}$ and N${\text{H}}_{4}^{+}$ were determined photometrically, and cultures were subcultivated in fresh medium after N${\text{O}}_{2}^{-}$ production ceased.

### Measurement of N_2_O Production in Enrichment Cultures

Nitrous oxide production was determined in triplicate 20 mL cultures supplemented with 0.5 mM NH_4_Cl, incubated in 120 mL glass serum bottles containing 21% oxygen in the headspace, and sealed with sterile butyl rubber stoppers and aluminum crimp caps. One additional vial containing only medium was incubated in parallel as negative control. Fifteen mL gas samples were collected at regular intervals, from which 12 mL were transferred into 10 mL sealed and evacuated glass vials, and stored at 4°C until analysis. In order to prevent vacuum formation in the cultivation vials, the volume of gas removed was replaced immediately with 15 mL filter-sterilized air. Gas analysis was performed by gas chromatography (AGILENT 6890N, Vienna, Austria; injector: 120°C, detector: 350°C, oven: 35°C, carrier gas: N_2_) connected to an automatic sample-injection system (DANI HSS 86.50, Headspace-Sampler, Sprockhövel, Germany). Nitrous oxide concentrations were determined with a ^63^Ni-electron-capture detector. Standard gases (Inc. Linde Gas, Vienna, Austria) contained 0.5, 1, and 2.5 μL/L N_2_O. A glass vial containing sterilized air was measured as control at each of five time-points.

### Scanning Electron Microscopy and DOPE-FISH Analyses

Cells for scanning electron microscopy were harvested from 2 mL of culture in late exponential N${\text{O}}_{2}^{-}$ production phase by centrifugation at 10,000 g and 4°C for 10 min, and processed as previously described (Abby et al., 2018) with slight modifications. Briefly, cells were resuspended in 1 mL 0.5% glutaraldehyde in 0.02 mM sodium cacodylate and incubated for 1 h, after which glutaraldehyde concentration was increased to 2.5% for 2 h. Fixed cells were washed three times in 0.02 mM sodium cacodylate and spotted onto 0.01%-poly-L-lysine coated glass slides (5 mm) and allowed to sediment for 15 min. Samples were dehydrated using a graduated ethanol series (30–100%, 15 min each) and dried by critical point drying (Leica EM CPD300). The slides were subsequently placed on conductive stubs, gold coated for 30 s (JEOL JFC-2300HR) and analyzed (JEOL JSM-IT200).

Cells for Doubly-labeled Oligonucleotide Probe Fluorescence *in situ* Hybridization (FISH) (Stoecker et al., 2010) were harvested from 2 mL culture by centrifugation at 4°C and 16,000 g for 40 min, and then washed in PBS-buffer and fixed with 4% paraformaldehyde for 3 h using standard protocols (Amann et al., 1990). Cells were washed two times with 1 mL PBS and finally resuspended in 200 μL of 1:1 PBS:ethanol mix before storage at −20°C. After dehydration in ethanol, cells were hybridized overnight in hybridization buffer containing 20% formamide using doubly-labeled probes Eub338 (Fluos) 5′-GCT GCC TCC CGT AGG AGT-3′ and Arch915 (dCy3) 5′-GTG CTC CCC CGC CAA TTC CT-3′.

### DNA Extraction

DNA for quantitative PCR (qPCR) and cloning PCR was extracted from cells collected from 1 to 2 mL culture by centrifugation at 4°C and 13,400 rpm for 30 min. DNA was extracted using a modified version of the protocol by Griffiths et al. (2000) and Urich et al. (2008), as follows: cells were resuspended in pre-warmed (65°C) 1% sodium dodecyl sulfate (SDS) extraction buffer and transferred to Lysing Matrix E tubes (MP Biomedicals) containing an equal volume of phenol/chloroform/isoamylalcohol (25:24:1). Cell lysis was performed in a FastPrep-24 (MP) device with speed setting 4 for 30 s, and the lysate was centrifuged at 13,400 rpm for 10 min. An equal volume of chloroform/isoamylalcohol (24:1) was added to the supernatant of the lysate, followed by centrifugation at 13,400 rpm for 10 min and collection of the aqueous phase. Nucleic acids were precipitated with an equal volume of isopropanol with addition of 40 μL NaCl 5 M and 1 μL glycogen (20 mg mL^−1^) as carrier, incubated for 1 h at room temperature. Following centrifugation at 13,400 rpm for 1 h, nucleic acid pellets were washed with 1 mL cold 70% ethanol, dried at 30°C using a SpeedVac centrifuge, eluted in nuclease-free water and stored at −20°C until analysis. Nucleic acid quantification for metagenome sequencing was performed with a Qubit™ 2.0 Fluorometer (Invitrogen) using the dsDNA HS Assay Kit.

### Quantitative PCR

Archaeal 16S rRNA genes were quantified in triplicate 20 μL reactions containing 10 μL GoTaq^®^ qPCR Master Mix 2x (Promega), 0.2 mg mL^−1^ BSA, 0.8 μM of each primer Cren-771F (5′-ACGGTGAGGGATGAAAGCT-3′) and Cren-957R (5′-CGGCGTTGACTCCAATTG-3′) (Ochsenreiter et al., 2003), and 2 μL DNA template. Amplification was performed with the following cycling conditions: 95°C for 10 min, followed by 40 cycles of 15 s denaturing at 95°C, 30 s joint annealing-extension at 54°C, extension at 60°C for 30 s, and fluorescence measurement at 78°C for 10 s. Standard dilutions were prepared in duplicates or triplicates ranging from 10 to 10^8^ gene copies μL^−1^ using long gene fragments from *N. viennensis* EN76 amplified with primers A109F (Großkopf et al., 1998) and A1492R (Nicol et al., 2008). The efficiency of qPCR assays ranged between 80 and 99% with R^2^ ≥ 0.99. Archaeal *amoA* genes were quantified in 20 μL reactions containing 10 μL GoTaq^®^ qPCR Master Mix 2x (Promega), 0.2 mg mL-1 BSA, 2 μL DNA template, and 1 μM of each of the primers CamoA-19F (5′-ATGGTCTGGYTWAGACG-3′) (Tourna et al., 2008; Pester et al., 2012) and the new primer TamoA-629R (5′-TGGCANTAYMGATGGATGGC-3′) (Arce et al., 2018), which was designed for improved coverage of archaeal *amoA* genes, particularly from *Ca*. Nitrosocosmicus spp. Amplification was performed using the following cycling conditions: 95°C for 10 min, followed by 40 cycles of 15 s denaturing at 95°C, 34 s joint annealing-extension at 58°C, extension at 60°C for 45 s, and fluorescence measurement at 78°C for 10 s. Standard dilutions were prepared in duplicates or triplicates ranging from 10 to 10^8^ gene copies μL^−1^ using genomic fragments containing the *amoA* gene of *Nitrososphaera viennensis* EN76 amplified with the new primers NV-LamoA-F (5′-CGCATGATCGGCCGCAGAGT-3′) and NV-LamoA-R (5′-GCCTAGTAGCGACCCGCCCT-3′), designed here specifically for *N. viennensis* EN76. The efficiency of qPCR assays ranged between 92 and 95%, with R^2^ ≥ 0.99. Bacterial 16S rRNA genes were quantified in 20 μL reactions containing 10 μL Sybr green Mix 2x (Qiagen), 0.2 mg mL^−1^ BSA, 0.5 μM of each of the primers P2 (5′-ATTACCGCGGCTGCTGG-3′) and P3 (5′-CCTACGGGAGGCAGCAG-3′) (Muyzer et al., 1993) and 2 μL DNA template. Amplification was performed with the following cycling conditions: 95°C for 15 min, followed by 40 cycles of 15 s denaturing at 95°C, 45 s joint annealing-extension at 45°C, and extension with fluorescence measurement at 72°C for 40 sec. Standard dilutions were prepared in duplicates or triplicates ranging from 10 to 10^8^ gene copies μL^−1^ using long gene fragments from *Nitrosospira multiformis* ATCC25196 amplified with primers Eubac27F and 1492R (Lane, 1991). The efficiency of all qPCR assays ranged between 98 and 100%, with R^2^ ≥ 0.99. Quantitative PCR assays were performed in a Mastercycler® ep gradient S realplex2 (Eppendorf AG), and the specificity of amplification products was confirmed by melting curve analysis and agarose gel electrophoresis.

### Archaeal *amoA* Gene Cloning and Sequencing

PCR amplification of archaeal *amoA* gene fragments (595 bp excluding primers) was performed in 50 μL reactions containing: 1.25 U of GoTaq® Flexi DNA Polymerase, 1 x Green GoTaq® Flexi Buffer (Promega, Madison, WI, USA), 2 mM MgCl_2_, 0.2 mM dNTPs and 1 μM of each primer CamoA-19F (5′-ATGGTCTGGYTWAGACG-3′) (Tourna et al., 2008; Pester et al., 2012) and TamoA-629R (5′-TGGCANTAYMGATGGATGGC-3′) (Arce et al., 2018). Thermal conditions were as follows: 5 min initial denaturing step at 95°C, followed by 35 cycles of 45 s denaturing at 95°C, 45 s annealing at 58°C and 45 s extension at 72°C, with a final extension step of 10 min at 72°C. Pooled triplicate PCR products were column-purified with the NucleoSpin® Extract II kit (Macherey-Nagel GmbH & Co. KG, Düren, Germany) according to the manufacturer's protocol and cloned in TOP10 chemically competent *Escherichia coli* cells using the TOPO TA Cloning® Kit for Sequencing (Invitrogen, Carlsbad, CA, USA). Clones were selected for sequencing after confirmation of the correct insert size by M13 colony-PCR and visualization on agarose gel electrophoresis. Plasmid extractions and sequencing of cloned sequences were processed by LGC Genomics (Berlin, Germany). Gene sequences have been deposited in GenBank under accession numbers MK978748–MK978767.

### 16S rRNA Gene Amplicon Sequencing and Analysis

PCR amplicons of 16S rRNA genes were obtained using primers 519F (5′-CAGCMGCCGCGGTAA-3′) (Burggraf et al., 1997) and 805R (5′-GACTACNVGGGTWTCTAAT-3′) (Apprill et al., 2015), which amplify both archaeal and bacterial genes. PCR amplicons were sequenced with Illumina MiSeq Paired-End sequencing (2x300 bp) at the Vienna BioCenter Core Facilities, Vienna. Data analysis was performed with QIIME2 (Caporaso et al., 2010). Adaptor sequences were first trimmed from reads using CUTADAPT (Martin, 2011) and then processed using the DADA2 pipeline (Callahan et al., 2016). Amplicon Sequence Variants (ASVs) were classified using VSEARCH (Rognes et al., 2016) with a custom-modified Silva v132 database.

### Genome Sequencing and Assembly

Sequencing reads were generated using two sequencing technologies: Illumina HiSeq 2500 generating 150 bp read pairs with a paired-end library with 900 bp insert size (VBCF sequencing facility, Vienna); and IonTorrent PGM 2015 with Chip 318C and 400 bp chemistry. DNA for Illumina sequencing was extracted from 41.5 mL culture (two pooled replicate cultures) and 1.3 μg were used for library preparation. DNA for IonTorrent sequencing was extracted from 150 mL culture (three pooled replicate cultures), and 196 ng were used for library preparation. In total, we obtained >50M read pairs through Illumina sequencing and 5.5M reads with IonTorrent. Reads were filtered by quality and length, and low complexity regions were removed. Illumina reads were filtered using Prinseq 0.20.4 (minimum length: 90, minimum quality: 28) (Schmieder and Edwards, 2011, RRID:SCR_005454). Filtering of IonTorrent reads (minimum length: 80, maximum length: 400 bp, minimum quality: 20) was performed using cutadapt 1.12 (Martin, 2011, RRID:SCR_011841). After filtering, we obtained 40M read pairs from the Illumina dataset and 4.6M reads from the IonTorrent dataset. All filtered reads were assembled using SPAdes 3.7 using kmer sizes from 11 to 105 by steps of 2 (Bankevich et al., 2012; RRID:SCR_000131). The filtered reads from both Illumina and IonTorrent were separately mapped onto the metagenomic assembly using NextGenMap 0.5.2 (Sedlazeck et al., 2013, RRID:SCR_005488). The read coverage of the SPAdes assembly was calculated with SAMtools 1.3.1 (Li et al., 2009, RRID:SCR_002105) for both IonTorrent and Illumina datasets. We then used the differential coverage binning approach (Albertsen et al., 2013) by plotting the coverages obtained using both sets of reads against each other, and using the % GC (Figure S5). Twenty-two scaffolds (27 contigs) above 5 kb were selected as belonging to the genome of *Ca*. N. arcticus Kfb. Five additional metagenomic bins were assigned to five distinct bacterial contaminants (Table S1). The genome sequence of *Ca*. N. arcticus Kfb has been deposited in NCBI under BioProject PRJNA505990 and BioSample SAMN10440610.

### Genome Annotation and Analysis

The 27 contigs were uploaded and annotated automatically using the Microscope annotation platform from Genoscope (Evry, France) (Vallenet et al., 2009; Medigue et al., 2017). Further annotations were obtained as in Kerou et al. (2016a) and Abby et al. (2018). The average nucleotide identity (ANI) between the three *Ca*. Nitrosocosmicus genomes was calculated based on whole-genome BLAST alignments using the Jspecies web server (Richter et al., 2016). Families of homologous proteins were built for each genome based on the results of a BLASTP run with the “all proteins against all” option. The results were used as input for the Silix (Miele et al., 2011) and Hifix (Miele et al., 2012) programs in order to cluster the sets of similar sequences into protein families. For sequences to be clustered in the same Silix family, they had to share at least 30% of identity and the BLAST alignment cover at least 70% of the two sequence lengths. We obtained a total of 4487 families of proteins for the *Ca*. Nitrosocosmicus genus.

Based on the genome bins from the SPAdes assembly (see subsection above) protein-coding genes were predicted using MetaGeneMark (Zhu et al., 2010) and rRNA gene identification was performed with RNAmmer (parameters *-S* bac *-multi -m* tsu, lsu, ssu) (Lagesen et al., 2007). Bacteria were taxonomically classified based on 16S and 23S rRNA genes using SILVA-ACT online (SINA 1.2.12 for ARB SVN revision 21565 accessed on 2018-07-31, minimum identity to query decreased to 0.6) (Pruesse et al., 2012), and based on inferred protein sequences using the Community Edition of MEGAN 6.10.13 (Huson et al., 2016) with LCA parameter top 5% and accession-to-taxonomy mapping file prot_acc2tax-Oct2017X1.abin and GenBank's non-redundant database (NRDB version 16/11/2017; BLASTP 2.7.1+ with E-value cutoff e-10). For a summary of the results see Table S1.

## Results and Discussion

### Enrichment and Cultivation of AOA From Arctic Soils

Two initial enrichment cultures of AOA were obtained from arctic mineral soils from a frost boil in a peatland and from upland moss tundra in Svalbard, by screening ammonia oxidation activity in approximately 100 initial cultures inoculated with diverse arctic soils, as described by Alves et al. (2013). Only archaeal ammonia oxidizers were detected in these enrichment cultures, based on analysis of *amoA* genes using archaea- and bacteria-specific PCR assays. After ~3 years of continuous cultivation, these ammonia-oxidizing cultures were shown to contain only a single AOA phylotype related to fosmid clone 29i4 (Quaiser et al., 2002), based on *amoA* and 16S rRNA genes (Alves et al., 2013), which is now represented by the candidate genus Nitrosocosmicus (Jung et al., 2016; Lehtovirta-Morley et al., 2016; Sauder et al., 2017). Therefore, we provisionally name here the newly enriched strain *Candidatus* Nitrosocosmicus arcticus strain Kfb (see detailed taxonomic characterization below).

**Figure 1:**
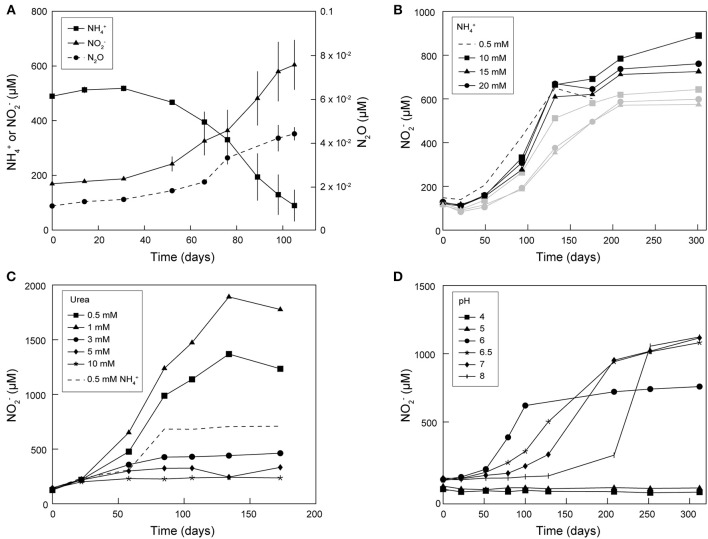
Enrichment cultures of *Ca*. N. arcticu*s* Kfb grown at 20°C under different conditions. **(A)** Cumulative net N${\text{H}}_{4}^{+}$ consumption, and net N${\text{O}}_{2}^{-}$ and N_2_O production in triplicate cultures. Error bars represent standard deviations. **(B–D)** Cumulative net N${\text{O}}_{2}^{-}$ production in enrichment cultures of *Ca*. N. arcticus Kfb under **(B)** different initial N${\text{H}}_{4}^{+}$ concentrations (black and gray lines represent cultures deriving from two distinct inocula, respectively), **(C)** different initial urea concentrations, and **(D)** different initial pH.

**Figure 2:**
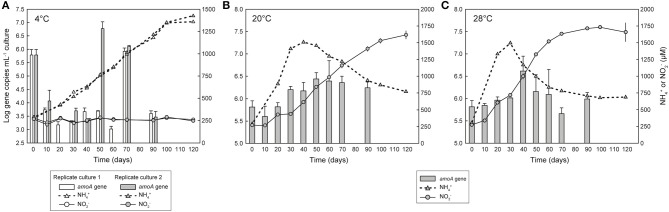
Growth of *Ca*. N. arcticus Kfb with or without nitrite production in enrichment cultures at **(A)** 4°C, **(B)** 20°C, or **(C)** 28°C. Cultures were grown in mineral medium supplemented with 1 mM urea. Growth was determined based on quantification of archaeal *amoA* gene copies with qPCR (gray bars). Solid and dotted lines represent cumulative net N${\text{O}}_{2}^{-}$ production and net N${\text{H}}_{4}^{+}$ release and consumption, respectively. **(A)** shows two replicate cultures with active growth but no N${\text{O}}_{2}^{-}$ production; the third replicate culture is not shown, as neither growth nor N${\text{O}}_{2}^{-}$ production were detected. Error bars in **(B,C)** represent the standard deviation of triplicate cultures; error bars in **(A)** represent triplicate qPCR reactions. Some error bars are smaller than the symbols and thus are not visible.

Continuous subcultivation of AOA enriched from frost boil soil over more than 5 years in mineral medium supplemented with antibiotics and NH_4_Cl or urea led to cultures with near- stoichiometric conversion of N${\text{H}}_{4}^{+}$ to N${\text{O}}_{2}^{-}$ at 20 and 28°C (Figures 1, 2). Ammonia oxidation activity was most stable at 20°C, as it tended to decrease or even stop in cultures continuously grown and subcultivated at 28°C. Therefore, most subsequent cultivation experiments were performed at 20°C. Ammonia oxidation rates were extremely slow in these cultures, with approximately 0.5 mM N${\text{H}}_{4}^{+}$ oxidized only after approximately 70 days at 20°C (Figure 1A). Maximal N${\text{O}}_{2}^{-}$ production after supplementation with NH_4_Cl was observed with a concentration of 10 mM, although only up to 0.9 mM N${\text{O}}_{2}^{-}$ was produced (Figure 1B). Supplementation with urea led to higher N${\text{O}}_{2}^{-}$ yields: up to 1 mM N${\text{O}}_{2}^{-}$ was produced with addition of 0.5 mM urea, and up to 1.9 mM N${\text{O}}_{2}^{-}$ with 1 mM urea (Figure 1C). When 3 mM urea was supplied, only a negligible amount of N${\text{O}}_{2}^{-}$ was produced, and only until day 50 of incubation. Ammonia oxidation was observed at pH 6–8 (with urea) at similar rates, although with increasingly longer lag phases at pH above 6 (up to 125 days at pH 8) (Figure 1D). Nitrous oxide (N_2_O) was produced in enrichment cultures of *Ca*. N. arcticus Kfb, but at very low concentrations of up to 0.04 μM (Figure 1A), consistent with the low N_2_O yields measured in cultures of *Nitrososphaera viennensis* EN76 and other AOA (Stieglmeier et al., 2014b; Kozlowski et al., 2016; Hink et al., 2017a,b). Like in cultures of other AOA, this N_2_O might have been mainly produced abiotically from NO leaked during ammonia oxidation (Kozlowski et al., 2016). Given the presence of other organisms in the enrichment cultures, the origin and mechanism of N_2_O production remains unclear.

Since growth rates with urea supplementation were suggestively faster and more stable than with NH_4_Cl, we used it as the substrate to assess ammonia oxidation over a temperature range of 4–32°C in parallel cultures, all inoculated with the same culture grown at 20°C. Urea was hydrolyzed to N${\text{H}}_{4}^{+}$ faster with increasing temperature (Figure S1A), although substantial ammonia consumption and N${\text{O}}_{2}^{-}$ production were only observed at 20 and 28°C, and to a much lesser extent at 16°C (Figures S1A,B). Different strategies were attempted to increase ammonia oxidation rates and the enrichment level of *Ca*. N. arcticus Kfb, namely through addition of different antibiotics (kanamycin, carbenicillin and ampicillin), lysozyme treatment, buffering the medium with HEPES, and supplementation with small amounts of pyruvate (0.5 mM). However, none of these strategies yielded faster ammonia oxidation rates or complete elimination of contaminant bacteria, with some treatments actually leading to inhibition of ammonia oxidation (i.e., media buffering with HEPES, or antibiotics other than streptomycin). We also observed an extension of the lag phase of ammonia oxidation when cultures were grown in volumes >20 mL, or when cultures had remained in stationary N${\text{O}}_{2}^{-}$ production phase for long periods before re-inoculation into fresh medium.

Based on analysis of 16S rRNA gene amplicons, *Ca*. N. arcticus Kfb has been enriched to between 72 and 93% of all organisms in the current enrichment cultures as of November 2018. All other organisms in the cultures represented uncultured bacterial strains, which, individually, comprised less than 10% of the community. Bacterial contaminants were affiliated with the genera *Devosia* and *Bradyrhizobium* (both class *Alphaproteobacteria*), *Acidimicrobium* (phylum Actinobacteria), and *Phycisphaera* (phylum Planctomycetes) (see Figure S5 and Table S1 for their corresponding genome bins).

### Ammonia Oxidation-Independent Growth of *Ca*. N. arcticus Kfb at Low Temperatures

Growth of *Ca*. N. arcticus Kfb, and initially possibly also other very closely-related AOA, was observed in several independent early enrichment cultures without concomitant oxidation of ammonia to N${\text{O}}_{2}^{-}$, but exclusively at 4 or 8°C, based on quantification of *amo*A and 16S rRNA genes. This behavior was reproduced in multiple cultures deriving from different cultivation lineages and at different enrichment stages, but originating from the same initial inoculum as the nitrifying culture of *Ca*. N. arcticus Kfb. However, this growth behavior was not consistent between all replicate incubations, nor in terms of incubation period and growth rates.

In an initial experiment we observed growth of AOA in two early enrichment cultures at 4°C based on comparable increases in both *amoA* and 16S rRNA gene copies, but without detectable N${\text{O}}_{2}^{-}$ production or N${\text{H}}_{4}^{+}$ consumption (Figure S2). These cultures derived from the same soil where *Ca*. N. arcticus Kfb was enriched from, and, consistently, at least one of them was dominated by *Ca*. N. arcticus Kfb-like organisms (i.e., nearly identical *amoA* genes) (see Supplementary Material for detailed results).

In order to confirm these observations, we investigated the ammonia oxidation-independent growth behavior over shorter time periods and compared it directly with growth associated with ammonia oxidation, by measuring growth of *Ca*. N. arcticus Kfb (based on *amoA* gene quantification) in triplicate cultures at 4, 8, 20, or 28°C supplemented only with 1 mM urea. All cultures were inoculated with the same ammonia-oxidizing culture grown continuously for several years at 20°C and supplemented only with urea. This culture derived directly from the same cultures from which the genome of *Ca*. N. arcticus Kfb was sequenced (see section below). Growth of *Ca*. N. arcticus Kfb was again detected in two out of three replicate cultures incubated at 4°C, and, consistent with previous observations, without detectable net N${\text{O}}_{2}^{-}$ production (Figure 2A). Cell numbers, inferred from *amoA* gene abundance, decreased drastically within the first 10 days of incubation, in contrast to ammonia-oxidizing cultures incubated at 20 or 28°C (Figures 2B,C). This possibly reflected an adaptive physiological and/or population response to the temperature shock, from 20°C in the culture used as inoculum to 4°C in this incubation. Cell numbers remained low but stable for 50 to 60 days, and were followed by a burst of growth within only 10 days, which far surpassed that observed in ammonia-oxidizing cultures throughout their whole incubation period. This period of fast growth was shifted by approximately 10 days between the two replicate cultures, possible as a result of different population dynamics during the temperature adjustment period. Like in cultures at 20 or 28°C, population sizes declined after reaching the abundance peak, although more abruptly. In contrast to cultures at 4°C, changes in *amoA* gene abundance between days 30, 50, and 70 in all replicate cultures incubated at 8°C (Figure S3) suggested only very limited growth, which was accompanied by production of small amounts of N${\text{O}}_{2}^{-}$ and thus could have been driven by ammonia oxidation.

Despite the limited reproducibility within individual experiments (i.e., among replicate cultures), growth of *Ca*. N. arcticus Kfb was repeatedly observed in the absence of detectable net oxidation of ammonia. This strongly suggests that this organism is able to conserve energy through an alternative metabolism(s), at least at 4°C. Although we could not identify potential alternative reductants, these results indicate that ammonia oxidation-independent growth did not depend on compounds present in the original soil, given that this behavior could be restored in cultures continuously transferred in artificial medium for several years. Since no exogenous organic compounds were added to the medium (only urea, vitamins, sodium bicarbonate, inorganic salts, chelated iron, and trace elements), growth likely depended on biomass recycling and/or interactions with other organisms or metabolites present in the enrichment cultures.

Such strategies and metabolic versatility—i.e., mixotrophy, facultative autotrophy, and utilization of both organic and inorganic electron donors—are common and widespread among microorganisms, as for example in Nitrospira (Watson et al., 1986; Gruber-Dorninger et al., 2015; Koch et al., 2015), Thiobacillus (Smith et al., 1980), and diverse hydrogen oxidizers (Piché-Choquette and Constant, 2019), such as Pseudomonas (Kiessling and Meyer, 1982), Thermomicrobium (Islam et al., 2019), and Mycobacterium (Greening et al., 2014).

### Growth Uncoupled From Ammonia Oxidation at Higher Temperatures

Remarkably, *amoA* gene quantification in ammonia-oxidizing cultures at 20 and 28°C revealed that growth of *Ca*. N. arcticus Kfb does not completely parallel ammonia oxidation dynamics, with most N${\text{O}}_{2}^{-}$ being produced before and after the periods of fastest cell growth (Figures 2B,C). In cultures at 20°C, N${\text{O}}_{2}^{-}$ concentrations increased almost linearly from day 10 to day 120 of incubation, except for a near-stationary N${\text{O}}_{2}^{-}$ production period between days 20 and 30 that coincided with the greatest increase in cell numbers (inferred from *amoA* gene abundance), and was followed by less pronounced growth between days 30 and 50 (Figure 2B). In cultures at 28°C, N${\text{O}}_{2}^{-}$ concentrations increased nearly exponentially from the beginning of incubation until day 50, and continued increasing at lower rates until day 100, although a substantial increase in cell numbers was only observed from day 30 to day 40 (Figure 2C). At both temperatures, approximately half of all N${\text{O}}_{2}^{-}$ was produced during the later incubation period alone (~50% of N${\text{O}}_{2}^{-}$ at 20°C and ~40% at 28°C), when cell numbers remained constant or even declined (Figures 2B,C). The growth behavior of *Ca*. N. arcticus Kfb in ammonia oxidizing cultures contrasts with that of other cultivated AOA, including other *Ca*. Nitrosocosmicus strains, which showed a tight correlation between N${\text{O}}_{2}^{-}$ production and growth (e.g., Tourna et al., 2011; Jung et al., 2016; Lehtovirta-Morley et al., 2016; Sauder et al., 2017). This apparent decoupling of growth and nitrite production also under temperatures at which ammonia oxidation was optimal further supports that *Ca*. N. arcticus Kfb is able to sustain growth through an alternative energy metabolism(s).

### Morphology

**Figure 3:**
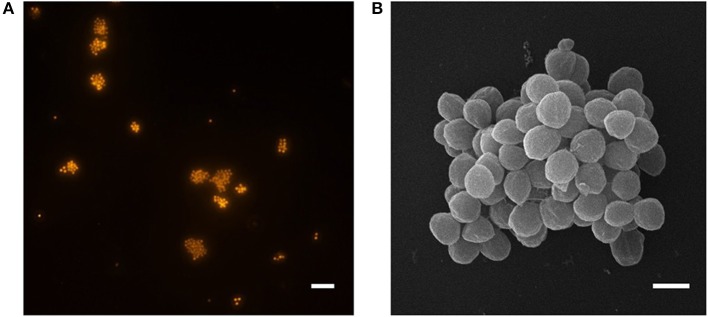
Micrographs of *Ca*. N. arcticus Kfb in enrichment cultures at 20°C. **(A)** DOPE-FISH micrograph using the archaea-specific probe ARCH 915, showing typical *Ca*. N. arcticus Kfb cell aggregates and occasional single cells. The scale bar represents 10 μm. **(B)** Scanning electron micrograph of a cell aggregate. Cells have an average diameter of 0.8 μm (ranging from 0.6 to 1.0 μm). The scale bar represents 1 μm.

*Ca*. N. arcticus Kfb was identified as coccoid-shaped cells of approximately 1 μm in diameter, based on FISH using archaea-specific dually labeled probes (DOPE-FISH). Figure 3 shows cells from an enrichment culture at late exponential N${\text{O}}_{2}^{-}$ production phase at 20°C (Figure 1A), where rod-shaped morphotypes were identified as bacteria (Figure S4). *Ca*. N. arcticus Kfb cells were found mainly in suspended aggregates typically comprising 10–20 cells, but often several more (Figures 3A,B and Figure S4). As observed by scanning electron microscopy, *Ca*. N. arcticu*s* Kfb cells are irregular cocci with an average diameter of 0.83 μm (standard deviation = 79 nm, range = 601–1070 nm, *n* = 115), which is slightly smaller than those of *Ca*. N. oleophilus MY3 (1.1 μm) (Jung et al., 2016), *Ca*. N. exaquare G61 (1.3 μm) (Sauder et al., 2017), and *Ca*. N. franklandus C13 (0.96 μm) (Lehtovirta-Morley et al., 2016) (Figure 3B and Table 1). *Ca*. N. arcticus did not exhibit the ridged walnut-like appearance observed in the closely related *Ca*. N. oleophilus MY3 (Jung et al., 2016), but had rather a relatively smooth spherical morphology more similar to that of *Ca*. N. exaquare G61. Nevertheless, these morphological differences could have resulted from the different cell fixation procedures in those studies, namely usage of PBS (phosphate-buffered Saline; 290 mOsm) and higher glutaraldehyde concentrations, which possibly caused extreme osmotic stress, loss of turgidity and lower hydration, leading to a wrinkled or collapsed cell appearance (Jung et al., 2016; Lehtovirta-Morley et al., 2016). Although *Ca*. N. arcticus Kfb occurred either as single cells or in clusters of varying size, like other *Ca*. Nitrosocosmicus strains (Figure 3A and Figure S4), cells were not covered by an apparent extracellular matrix, similar to that observed in cell clusters of *Ca*. N. exaquare G61 and *Ca*. N. oleophilus MY3 (Jung et al., 2016; Sauder et al., 2017).

**Table 1:** Characteristics of *Ca*. N. arcticus Kfb and other cultivated members of candidate genus Nitrosocosmicus.

**Organism**	**Source**	**Status**	**Avg. size (μm)**	**Growth temp. optimum (range) (^**°**^C)**	**pH optimum (range)**	**Maximum specific growth rate (μmax, h^**−1**^)**	**Specific NO prod. rate (fmol ${\text{NO}}_{2}^{-}$ cell^**−1**^ h^**−1**^)**	**Specific ${\text{NO}}_{2}^{-}$ prod. rate at μmax (fmol ${\text{NO}}_{2}^{-}$ cell^**−1**^ h^**−1**^)**	**Inhib. ${\text{NH}}_{4}^{+}$ conc. (mM)**	**Stimulation by organic compounds**	**Genome size (Mb)**	**DNA G+C (mol %)**	**No. protein-coding genes**	**16S/23S/5S rRNA gene copies**	**GOGAT subunits**	**Reference**
*Ca*. N. arcticus Kfb	Frost boil in arctic tundra fen (mineral gleysol)	Enriched	1.0	4 (4–8)^a^ 28 (20–28)^b^	6 (6–7)	0.028–0.033 (4°C) 0.004–0.012 (20°C) 0.002–0.010 (28°C)	0.09–0.74 (20°C)^d^ 0.19–0.86 (28°C)^d^	−0.07–0.04 (4°C)^e^ 0.01–0.74 (20°C)^e^ 0.19–0.61 (28°C)^e^	>20	Yeast extract (suggestive)	2.65^i^	34.0%	3104	3/3/1	NARC_80135—NARC_80138	This study
*Ca*. N. oleophilus MY3	Hydrocarbon-contaminated terrestrial sediment	Pure	1.1	30 (20–35)	6.5–7 (5.5–8.5)	0.013	n. a.	n. a.	50	yes^f^	3.43	34.1%	3725	3/3/1	NMY3_03179—NMY3_03182	(Jung et al., 2016)
*Ca*. N. exaquare G61	Wastewater treatment plant (biofilm)	Enriched	1.3	33 (21–40)	8^c^	n. a.	n. a.	n. a.	20	yes^g^^,^ ^h^	2.99	33.9%	3162	2/2/1	–	(Sauder et al., 2017)
*Ca*. N. franklandus C13	Agricultural sandy loam soil	Pure	0.96	40 (30–45)	7 (6–8.5)	0.024	0.58	n. a.	>100	n. a.	n. a.	n. a.	n. a.	n. a.	–	(Lehtovirta-Morley et al., 2016)

*n. a., not available*.

a *Non-nitrifying*.

b *Nitrifying*.

c *Default growth pH; growth range not reported*.

d *Calculated based on all time periods measured during exponential N${\text{O}}_{2}^{-}$ production*.

e *All growth parameters of Ca. N. arcticus Kfb at different temperatures are based on parallel cultures derived from the same inoculum; values were calculated from duplicate cultures at 4°C (where growth was observed), quadruplicate cultures at 20°C, and triplicate cultures at 28°C*.

f *Stimulated slightly by fructose, glucose, arabinose, peptone, yeast extract, and casamino acids*.

g *Stimulated by malate, succinate, pyruvate, citrate, butyrate, glucose, glycerol, acetate, taurine, and yeast extract*.

h *Inferred based on N${\text{O}}_{2}^{-}$ production, not actual cell growth or biomass increase*.

i *genome not closed; 23 contigs*.

The typical formation of large cell aggregates indicates that *Ca*. N. arcticus Kfb, like other *Ca*. Nitrosocosmicus strains, is able to form biofilm-like structures, which is also supported by the presence of genes encoding exopolymeric substances (EPS) in the genomes of all *Ca*. Nitrosocosmicus strains (see also genome analyses), as well as observed formation of a putative extracellular matrix by *Ca*. N. oleophilus MY3 and *Ca*. N. exaquare G61 (Jung et al., 2016; Sauder et al., 2017). Moreover, cell aggregates of *Ca*. N. arcticus Kfb were resistant to sonication, indicating that these associations are very stable and occur naturally rather than resulting from technical artifacts, and thus might have an important role in the organism's physiology and life-style.

### Taxonomy and Environmental Distribution

The 16S rRNA gene of *Ca*. N. arcticus Kfb shares 100%, 99.7%, and 99.3% sequence identity with those of *Ca*. N. oleophilus MY3, *Ca*. N. exaquare G61, and *Ca*. N. franklandus C13, respectively, whereas its *amoA* gene shares 96.7% sequence identity with that of *Ca*. N. oleophilus MY3 and 91% with both later strains (all comparisons are based on full-length genes, except those with *Ca*. N. franklandus C13, for which only near-full length gene fragments are available). Despite harboring identical 16S rRNA genes, the genomes of *Ca*. N arcticus Kfb and *Ca*. N. oleophilus MY3 are surprisingly divergent and share only 83% average nucleotide identity (ANI) (Konstantinidis et al., 2006) over the 64% fraction of *Ca*. N arcticus Kfb's genome that could be aligned (see Methods and genome analysis below). The ANI between the genomes of *Ca*. N. arcticus Kfb and *Ca*. N. exaquare G61 is 73%, based on 47% of the genome aligned. These ANI values are well below the threshold of <94% proposed to represent different species (Goris et al., 2007; Richter and Rossello-Mora, 2009), thus supporting the proposal of *Candidatus* Nitrosocosmicus arcticus strain Kfb as a new species within the candidate genus Nitrosocosmicus. The main characteristics of *Ca*. N. arcticus Kfb compared to other currently cultivated *Ca*. Nitrosocosmicus strains are summarized in Table 1. The highly conserved 16S rRNA gene sequences (100% similarity between *Ca*. N. arcticus and N. oleophilus MY3) but large differences in their genomes (see below) and habitats indicate that *Ca*. Nitrosocosmicus strains appear to evolve fast and experience unusually frequent horizontal gene transfer.

**Figure 4:**
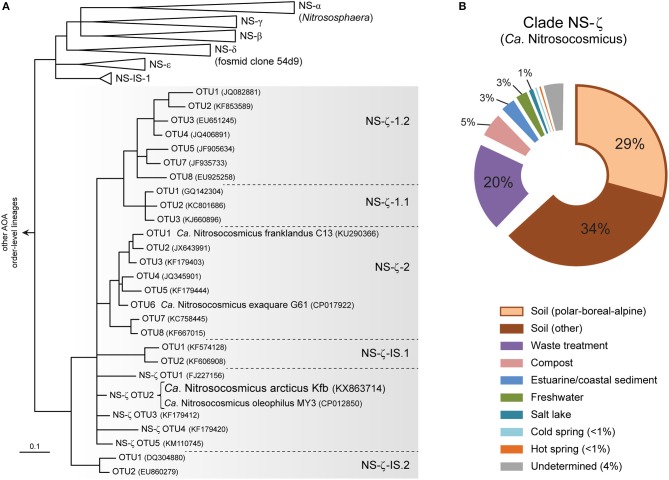
Phylogeny and environmental distribution of AOA clade NS-ζ (Zeta), representing the candidate genus Nitrosocosmicus based on *amoA* genes. **(A)** Tree showing the *amoA* gene diversity within clade NS-ζ and the phylogenetic placement of *Ca*. N. arcticus Kfb and other cultivated *Ca*. Nitrosocosmicus species. Only the order-level *amoA* lineage NS (*Nitrososphaerales)* is shown, [adapted from the publicly-available reference phylogeny by Alves et al. (2018)]. Organisms and OTUs not associated with a specific subclade are classified as a basal NS-ζ OTU (e.g., *Ca*. N. arcticus Kfb is classified as NS-ζ-OTU2). All branches are highly supported and OTUs defined at 96% sequence identity (Alves et al., 2018). **(B)** Global environmental distribution of clade NS-ζ based on 364 *amoA* genes in the curated database (Alves et al., 2018). Sequences from polar, boreal and alpine soils and from cold springs were further categorized here.

Based on a recent global phylogeny-guided taxonomy of archaeal *amoA* genes by Alves et al. (2018) the candidate genus Nitrosocosmicus represents AOA clade NS-ζ (NS-Zeta) within the order-level lineage NS (i.e., order *Nitrososphaerales*) (Figure 4A), which was previously referred to as “*Nitrososphaera*-sister cluster” (Pester et al., 2012). *Ca*. N arcticus Kfb is specifically associated with one of several basal OTUs within clade NS-ζ, which also includes the two major subclades NS-ζ-1 (without cultivated organisms), and NS-ζ-2, represented by *Ca*. N. exaquare G61 and *Ca*. N. franklandus C13 (Figure 4A and Figure S6).

Based on environmental studies of *amoA* genes, clade NS-ζ has been found mainly in soils, although it is also particularly frequent in wastewater treatment plants (Figure 4B; Alves et al., 2018). Additionally, it has also been detected, albeit to less extent, in compost, estuarine and coastal sediments, salt lakes and also in both hot and cold terrestrial springs.

### Genome Analyses and Metabolic Predictions

The genome of *Ca*. N. arcticus Kfb was assembled from enrichment culture using a whole-genome shotgun sequencing approach with both Illumina HiSeq and IonTorrent methods (see Methods). The 2.7 Mb genome has an estimated completeness of 98%, comprising 22 genomic scaffolds (27 contigs), based on analysis of 145 lineage-specific marker genes (which yielded 98% and 99% genome completeness for the other two *Ca*. Nitrosocosmicus strains as well). Based on the high coverage of the rRNA gene regions in our assembly, *Ca*. N. arcticus Kfb appears to encode three rRNA gene operons, similar to other *Ca*. Nitrosocosmicus strains (Table 1). However, 16S and 23S rRNA gene coding sequences were located in individual contigs, and thus we were unable to confirm the occurrence of multiple rRNA operons within the genome context. Interestingly, none of the other Thaumarchaeota or archaea of the TACK superphylum have more than one rRNA operon. Multiple rRNA operons have been associated with higher growth rates and faster response to environmental changes, including resource availability (Klappenbach et al., 2000; Roller et al., 2016), which may reflect fundamental physiological differences between *Ca*. Nitrosocosmicus spp. and other AOA characterized to date.

**Figure 5:**
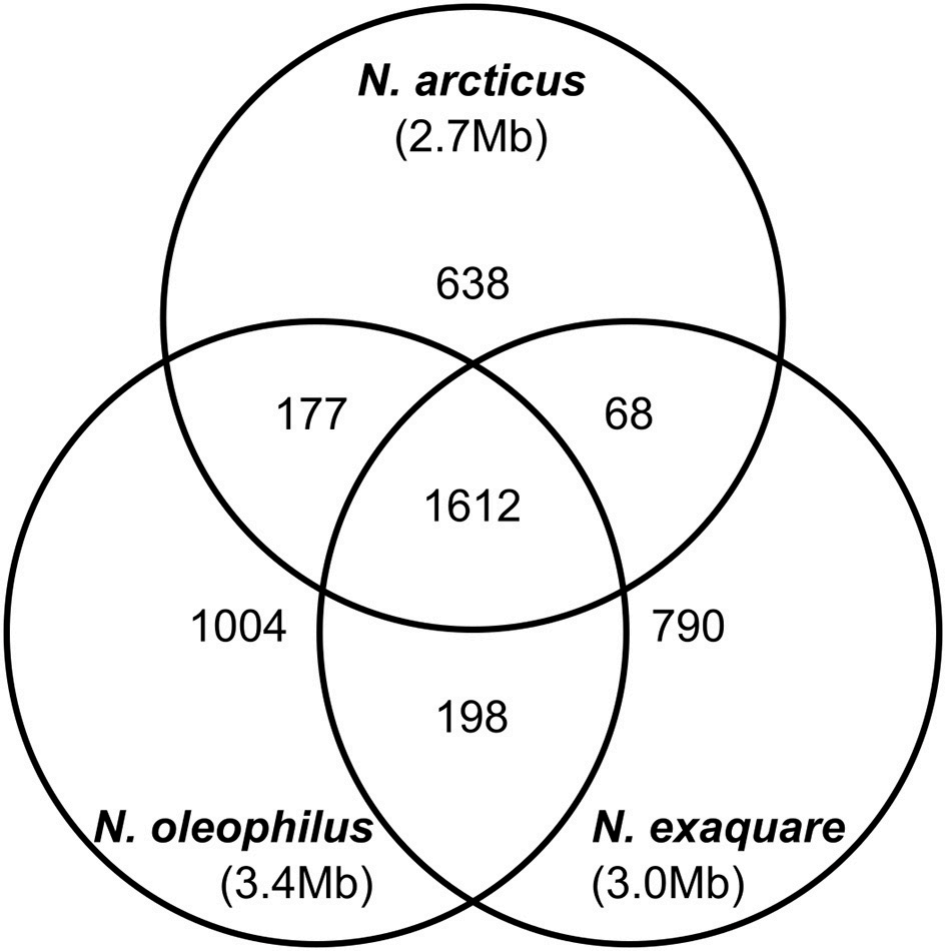
Genomic comparison of strains from candidate genus Nitrosocosmicus. The Venn diagram shows the distribution of homologous protein families among the three *Ca*. Nitrosocosmicus genomes available, as well as their genome sizes (see text for details).

*Ca*. N. arcticus Kfb has the full gene sets encoding the ammonia-monooxygenase (AMO), urease and the 3-hydroxypropionate/4-hydroxy-butyrate pathway for carbon fixation, characteristic of all studied AOA (see annotations, Table S2). In order to compare the protein-coding gene complements of the three *Ca*. Nitrosocosmicus genomes available, we determined families of homologous genes based on protein sequence comparison (see Methods for details) and assessed their distribution among the three genomes (Figure 5). *Ca*. N. arcticus Kfb has the smallest genome within the genus and encodes 638 strain-specific homologous protein families (26% of its families), whereas *Ca*. N. oleophilus MY3 and *Ca*. N. exaquare G61 each encode 34% and 30% strain-specific protein families, respectively, proportional to their larger genomes. As expected from their phylogenetic relationships (Figure 4A and Figure S6), *Ca*. N. arcticus Kfb shares more protein families with *Ca*. N. oleophilus MY3 than with *Ca*. N. exaquare G61 (177 vs. 68 protein families, respectively). The protein coding density of *Ca*. N. arcticu*s* Kfb genome is approximately 75%, whereas those of *Ca*. N. oleophilu*s* MY3 and *Ca*. N. exaquare G61 are 74% and 77%, respectively. Collectively, *Ca*. Nitrosocosmicus genomes have the lowest coding density among AOA [e.g., 86% for *N. viennensis* EN76, and 92% for *N*. maritimus SCM1; data from Microscope (Medigue et al., 2017)].

**Figure 6:**
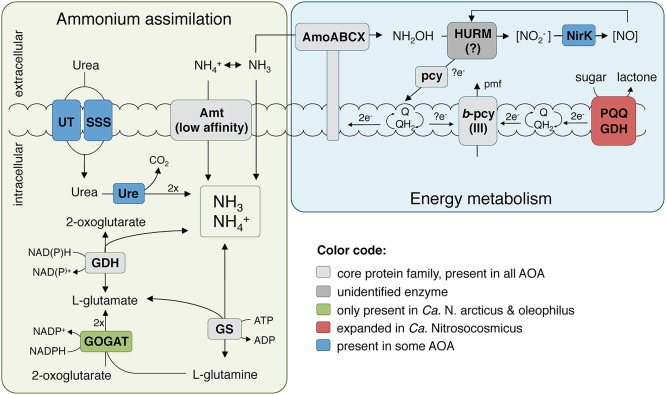
Reconstruction of the putative energy metabolism and primary nitrogen assimilation pathways in *Ca*. N. arcticus Kfb. The ammonia oxidation pathway representation was adapted from Kozlowski et al. (2016), Abbreviations: AMO, ammonia monooxygenase; Amt, ammonium transporter family; GDH, glutamate dehydrogenase; GOGAT, glutamate synthase; GS, glutamine synthetase; HURM, putative hydroxylamine:ubiquinone redox module; NirK, Cu-containing nitrite reductase; pcy, plastocyanin; pmf, proton-motive force; PQQ GDH, PQQ-dependent glucose/sorbosone dehydrogenase; Q/QH_2_, quinone/quinol pool; Ure, urease holoenzyme; UT, urea transporter family; SSS, solute:sodium symporter family.

The presence of a full 3-hydroxypropionate/4-hydroxybutyrate carbon fixation pathway and all genes for gluconeogenesis indicates that *Ca*. N. arcticus Kfb is in principle capable of autotrophic growth like other AOA strains (Könneke et al., 2014; Kerou et al., 2016b). The organism can also produce polyhydroxyalcanoates as carbon storage compounds (Poli et al., 2011) and compatible solutes (mannosylglycerate) (Empadinhas and da Costa, 2008), like several other AOA. *Ca*. N. arcticus Kfb also encodes two carbonic anhydrases, enabling it to interconvert between bicarbonate and CO_2_ (Ferry, 2010), as found in several other marine and terrestrial lineages of AOA. The genome encodes six members of the glucose/sorbosone dehydrogenase family proteins (PF07995, arCOG02796), a protein family generally expanded among *Ca*. Nitrosocosmicus spp. in comparison to other AOA (9–17 found in *Ca*. Nitrosocosmicus spp. vs. 3–6 in *Nitrososphaera* spp. vs. 2–3 in *Nitrosopumilales* spp.). These periplasmic or membrane bound pyrroloquinoline quinone (PQQ)-dependent proteins are known to oxidize aldose sugars into their corresponding lactones by simultaneously reducing a variety of electron acceptors, such as cupredoxins or quinones (Toyama et al., 2004), and thereby contributing reducing equivalents to the respiratory chain (Figure 6). A more thorough characterization of this expanded family of dehydrogenases in *Ca*. N. arcticus Kfb and related strains might provide clues regarding their peculiar growth behavior independent of ammonia oxidation. For instance, potential growth substrates (e.g., sugars, alcohols, organic acids) could derive from cellular components or metabolites produced during growth and population turnover of the various organisms in the enrichment cultures. In addition, *Ca*. N. arcticus Kfb and all currently analyzed *Ca*. Nitrosocosmicus strains encode putative beta-1,2-mannosidases (CAZy family GH130), which are enzymes involved in the degradation of mannans originating from plant cell walls (Cuskin et al., 2015; Nelkner et al., 2019). The resulting sugars could either serve as substrates for the PQQ-dependent glucose/sorbosone dehydrogenases or be further transported into the cell by a yet-unidentified sugar transporter and enter the central carbon metabolism (even though it is yet unclear if AOA can perform glycolysis).

Uniquely among currently analyzed genomes of thaumarchaea, all *Ca*. Nitrosocosmicus strains encode the full gene set for molybdenum cofactor (Moco) biosynthesis (Mendel and Leimkühler, 2015) and a putative ABC type molybdate/tungstate transporter (Hagen, 2011). All *Ca*. Nitrosocosmicus strains also encode two copies of a periplasmic DMSO/TMAO reductase protein superfamily (arCOG00266), one of which is adjacent to the Moco biosynthesis gene cluster. The substrate of the thaumarchaeal homologs is unknown, but given their taxonomic distribution (highest abundance and diversity in environmental bacteria) and roles of characterized members of this superfamily, they could be involved in energy metabolism or in detoxification processes (Leimkühler and Iobbi-Nivol, 2016). It is noteworthy that two of the characterized *Ca*. Nitrosocosmicus species were isolated from contaminated environments (i.e., coal-tar contaminated sediments, and wastewater), where also the highest diversity of DMSO/TMAO reductases has been found (Leimkühler and Iobbi-Nivol, 2016).

Surprisingly, *Ca*. N. arcticus Kfb encodes the four subunits of the archaeal type of glutamate synthase (GOGAT), which are also present in the closely-related *Ca*. N. oleophilus MY3 but not in *Ca*. N. exaquare G61 or other AOA. GOGAT catalyzes the NADPH-dependent formation of two glutamate molecules from glutamine and 2-oxoglutarate, which, together with the ATP-dependent glutamine synthetase (GS), constitutes a high-affinity and energy-consuming ammonia assimilation pathway that functions under low ammonia concentrations, when the cell is not limited for energy and carbon (Helling, 1994) (Figure 6). This may provide further evidence that *Ca*. Nitrosocosmicus spp. have an alternative or auxiliary energy metabolism, possibly more efficient than ammonia oxidation, allowing them to invest energy on ammonia assimilation under favorable environmental conditions. Conversely, the absence of GOGAT in other AOA implies that they assimilate ammonia primarily via glutamate dehydrogenase (GDH), a low-affinity enzyme that catalyzes the reversible reductive amination of 2-oxoglutarate to glutamate. The dominant view is that this route is active under energy and carbon limitation, but ammonia excess conditions, because no ATP is consumed and less carbon is used per ammonia molecule assimilated (Helling, 1994). This is the dominant pathway in hyperthermophilic archaea (Robb et al., 2001) and haloarchaea (Bonete et al., 2008) for assimilation of ammonia directly supplied in non-limiting growth medium, as opposed to ammonia originating from amino acid catabolism or nitrate reduction. Moreover, it has been shown that energy and carbon limitation stimulate GDH expression in some bacteria even under nitrogen limiting conditions (van Heeswijk et al., 2013). These observations may explain its preferential use in all other AOA that are rather considered to operate under energy limiting and oligotrophic conditions (Prosser and Nicol, 2012), as this enzyme provides a direct link, and therefore a convenient point of regulation, between carbon and nitrogen metabolism.

*Ca*. Nitrosocosmicus genomes also encode the highest number of multicopper oxidase family proteins among thaumarchaea (6–8 vs. 2–5 in all others), with three families being specific to *Ca*. N. arcticus Kfb, out of a total of six in the genome. As observed in other *Ca*. Nitrosocosmicus strains, *Ca*. N. arcticus Kfb encodes a peroxiredoxin and a Mn-catalase, which confer resistance to reactive oxygen species and therefore constitute a successful adaptation to an aerobic terrestrial environment (Jung et al., 2016; Sauder et al., 2017). *Ca*. N. arcticus Kfb also encodes an extended repertoire of genes responsible for acetamidosugar biosynthesis, glycosylation and extracellular polysaccharide production, similar to *Ca*. N. oleophilus MY3 (Jung et al., 2016), which indicates an ability to form extracellular polymeric substances and biofilms. Among the protein families specific to *Ca*. N. arcticus Kfb, we also found seven families of integrases/recombinases and a putative provirus, indicating an actively mobile genome.

## Conclusions

*Ca*. N arcticus Kfb represents the first ammonia oxidizing archaeon enriched from a terrestrial arctic environment. This organism grows unusually slow as an ammonia oxidizer, in comparison to other cultivated AOA strains, suggesting that current growth conditions in greater potential cultures are sub-optimal. Nevertheless, apparently faster growth rates, within the range of other AOA cultivated under ammonia-oxidizing conditions, have been repeatedly observed at lower temperatures without detectable N${\text{O}}_{2}^{-}$ production (see Table 1). These observations indicate that the organism is able to grow mixotrophically and/or based on a primary energy metabolism other than ammonia oxidation, probably using organic compounds. In line with this hypothesis, we have identified a number of common features in the genomes of *Ca*. N. arcticus and other *Nitrosocosmicus* strains that indicate for organotrophic or heterotrophic growth than other characterized AOA. However, despite numerous attempts, we could not identify alternative electron donors or acceptors of *Ca*. N. arcticus, or the optimal conditions for this potential alternative metabolism, mainly due to the slow and erratic growth behavior in culture, as well as the presence of multiple bacterial contaminants. Growth and/or ammonia oxidation of at least two of the three other cultivated *Ca*. Nitrosocosmicus strains (*Ca*. N. oleophilus MY3 and *Ca*. N. exaquare G61) have indeed been shown to be strongly stimulated by organic compounds, further suggesting that organisms from this lineage might be generally able to grow organotrophically or mixotrophically, by complementing their energy requirements using alternative reductants.

Interestingly, the apparent alternative growth mode of *Ca*. N. arcticus Kfb without ammonia oxidation was most consistently induced by low temperatures similar to those experienced by the organism in its natural habitat. Due to very low external inputs, ammonium bioavailability in arctic soils depends mainly on mineralization of organic matter, which is severely limited by chronic low temperatures, despite the often-large amounts of organic material present. While it is unknown if a temperature-dependent functional switch can also be induced in other *Ca*. Nitrosocosmicus strains, it is tempting to speculate that *Ca*. N. arcticus Kfb may have an enhanced alternative or auxiliary metabolism, in relation to that of the closely-related *Ca*. N. oleophilus MY3 (and possibly other AOA), as part of a more complex adaption to its native cold, low ammonium and highly organic environment.

Moreover, the growth and ammonia oxidation dynamics over the temperature range tested suggest that this organism increasingly depends on ammonia oxidation for growth at higher temperatures, but possibly as a stress response and/or backup mechanism to support growth under suboptimal temperature conditions. Such functional switch by arctic soil AOA can, in turn, have potentially large implications for nitrogen cycling in these ecosystems. Our future investigations will be based on the hypothesis that these AOA populations are primarily sustained by organotrophic metabolism under the native low temperatures, but switch increasingly to ammonia oxidation under higher temperatures, thus fueling higher nitrification and denitrification rates, and potentially also higher N_2_O emissions.

We propose a *Candidatus* status for the thaumarchaeal strain cultivated here, with the following taxonomic assignment:

- Class *Nitrososphaeria*
- Order *Nitrososphaerales*
- Family *Nitrososphaeraceae*
- *Candidatus* Nitrosocosmicus arcticus sp. nov. strain Kfb.

Etymology: L. adj. nitrosus, “full of natron,” here intended to mean nitrous (nitrite producer); L. masc. n. cosmicus, cosmopolitan; arcticus (L. masc. gen) describes origin of sample (arctic soil).

Source: arctic mineral soil from a frost boil in a tundra fen peatland in Knudsenheia, Svalbard, Norway (Alves et al., 2013).

Description: a facultative ammonia-oxidizing archaeon of the phylum Thaumarchaeota, able to utilize ammonium and urea as substrates for ammonia oxidation at temperatures between 16 and 28°C, at pH ranging from 6 to 7; spherically shaped with a diameter of 1 μm.

Differentiation relative to closest relative: the 16S rRNA and *amo*A genes are 100 and 96.7% identical to those of *Ca*. N. oleophilus MY3, respectively. Based on average nucleotide identity (ANI) between all *Ca*. Nitrosocosmicus strains (<94%) and extensive differences in protein-coding gene complement, we propose a separate species name for strain Kfb.

## Data Availability

The genome sequence of *Ca*. N. arcticus Kfb has been deposited in the NCBI database under BioProject PRJNA505990. Cloned *amoA* gene sequences have been deposited in GenBank under accession numbers MK978748–MK978767.

## Author Contributions

RA set up and characterized enrichment cultures, analyzed growth properties, and discovered uncoupled growth. MK analyzed genome features. AZ characterized growth conditions and assembled the genome. RB maintained and repeatedly rescued strain in culture over 6 years. SA and HS performed bioinformatic analyses and genome assembly. KP did electron and phase contrast microscopy. CS conceived the study and wrote the first and final drafts of the manuscript. RA, MK, and CS interpreted results and wrote the manuscript with contributions from all co-authors.

### Conflict of Interest Statement

The authors declare that the research was conducted in the absence of any commercial or financial relationships that could be construed as a potential conflict of interest.
