# Supplementary material for: Ammonia Oxidation by the Arctic Terrestrial Thaumarchaeote Candidatus Nitrosocosmicus arcticus Is Stimulated by Increasing Temperatures
Source: Front Microbiol. 2019 Jul 17;10:1571. doi: 10.3389/fmicb.2019.01571 (PMC6657660; doi:10.3389/fmicb.2019.01571)
Supplement: Supplementary file 1 [file Data_Sheet_1.PDF]

SUPPLEMENTARY MATERIAL

Ammonia oxidation by the arctic terrestrial thaumarchaeote *Ca*. Nitrosocosmicus arcticus is stimulated by increasing temperatures

Ricardo J. Eloy Alves[1†], Melina Kerou[1], Anna Zappe[1,2], Romana Bittner[1], Sophie S. Abby[1‡], Heiko A. Schmidt[2], Kevin Pfeifer[1,3], and Christa Schleper[1]*

[1]Archaea Biology and Ecogenomics Division, Department of Ecogenomics and Systems Biology, University of Vienna, Althanstrasse 14, 1090 Vienna, Austria; [2]Center for Integrative Bioinformatics Vienna (CIBIV), Max F. Perutz Laboratories, Medical University of Vienna, University of Vienna, Campus Vienna Biocenter 5 (VBC5), 1030 Vienna, Austria; [3] Institute for Synthetic Bioarchitectures, University of Natural Resources and Life Sciences, Vienna, Austria

[†]Present address: Climate and Ecosystem Sciences Division, Earth and Environmental Sciences, Lawrence Berkeley National Laboratory, Berkeley, CA, USA

[‡]Present address: Université Grenoble Alpes, Centre National de la Recherche Scientifique (CNRS), Laboratoire Techniques de l'Ingénierie Médicale et de la Complexité - Informatique, Mathématiques et Applications, Grenoble (TIMC-IMAG), Grenoble, France.

SUPPLEMENTARY RESULTS

Ammonia oxidation-independent growth of *Ca.* N. arcticus Kfb at low temperatures

In a first experiment, we used as inoculum two enrichment cultures where AOA growth had been observed at 4°C without ammonia oxidation, each deriving from distinct arctic soil types, i.e., a peatland frost boil in Knudsenheia (Kfb, same soil inoculum as for ammonia-oxidizing cultures) and upland moss tundra in Longyearbyen (Lmt) (Alves et al. 2013). These cultures were each used to inoculate two new subcultures incubated at 4°C in the same medium containing 0.5 mM NH_4^+, but with yeast extract (0.01%) added to one of them. Growth of AOA was observed in both Kfb subcultures based on comparable increases in both *amoA* and 16S rRNA gene copies, although without net NO_2^- production or NH_4^+ consumption in either culture (Figure 2). A very small amount of NO_3^- was produced throughout the 110-day incubation period (~20 mM), likely due to the presence of nitrite-oxidizing bacteria in these early enrichments, and a substantial amount of NH_4^+ was produced through nitrogen mineralization in the culture containing yeast extract. Copies of both *amoA* and 16S rRNA genes increased progressively in both cultures from day 20 to days 60 and 80, representing final cell increases of approximately one and two orders of magnitude in cultures Kfb.A1 (Figure 2A) and Kfb.A2 (Figure 2B), respectively. Notably, 19 out of 20 cloned *amoA* sequences (95%) obtained at day 80 from the culture with greatest growth (i.e., culture Kfb.A2) were nearly identical to that of *Ca.* N. arcticus Kfb (≥99% sequence identity, sequences are available in GenBank under accession numbers MK978748- MK978767), contrary to the original inoculum culture, where *Ca.* N. arcticus Kfb-like AOA accounted for only 6% of *amoA* genes (clade C in (Alves et al. 2013)), now included in clade NS-ζ (Alves et al. 2018). In contrast, neither ammonia oxidation activity nor growth of AOA were observed in Lmt cultures (*amoA* and 16S rRNA gene copy numbers remained constant or decreased), at least between the two time-points analyzed (days 20 and 60), in spite of *Ca.* N. arcticus Kfb-like AOA comprising a greater fraction of AOA in the Lmt inoculum culture (47%) than in that of Kfb subcultures (6%) (Alves et al. 2013) (data not shown). Growth at low temperature (8°C) independent of ammonia oxidation was successfully reproduced in two out of three replicate cultures, at a later enrichment stage of *Ca.* N. arcticus Kfb, supplemented only with urea, although over a very long incubation period (~180 days) (not shown)

SUPPLEMENTARY TABLES AND FIGURES

Table S1

Characteristics, classification, coverage and relative enrichment of the genomes obtained from Ca. N. arcticus Kfb enrichment cultures.

Organism	No. scaffolds	Total scaffold length (Mb)	Scaffold mean %GC	RNAmmer predictions[a] (16S, 23S, 5S)	MEGAN classification[b]	SILVA classification[c] (SSU/LSU)	SILVA identity [%][c] (SSU/LSU)	Normalized coverage	Relative enrichment
Ca. N. arcticus Kfb	23	2651882	34.03	+				1767.57	44.73%
contaminant 1	11	487735	62.63	-	*Devosia* sp. 66-22 (species)	*not available*	-	948.07	23.99%
contaminant 2	35	4041616	63.69	+	*Devosia* sp. 66-22 (species)	*Devosia* (genus)	97.15 / 84.50	525.00	13.29%
contaminant 3	42	4855308	63.30	+	*Devosia* (genus)	*Devosia* (genus)	97.58 / 84.18	418.50	10.59%
contaminant 4	63	6404212	63.21	+	*Bradyrhizobiaceae* (family)	*Bradyrhizobium* (genus)	99.01 / 94.79	258.64	6.55%
contaminant 5	7	3846127	70.81	+	*Actinobacteria* (phylum)	*Acidimicrobiia* (class)	94.80 / 84.41	33.63	0.85%

[a] RNAMMER was run with parameters -S bac -multi -m tsu,lsu,ssu

[b] Classification based on predicted protein sequences matched against GenBank's NRDB (version 16/11/2017); MEGAN was run with top 5% in the LCA parameters

[c] SILVA-ACT was run online (SINA v1.2.12 for ARB SVN revision 21565) with classification enabled for the respective molecule class (SSU/LSU) with default parameters, except with min. identity to query decreased to 0.6

Table S2. Annotation of genes and function in the genomic sequence of *Ca.* N. arcticus Kfb.

Provided as separate XLSX file under Supplementary Material.

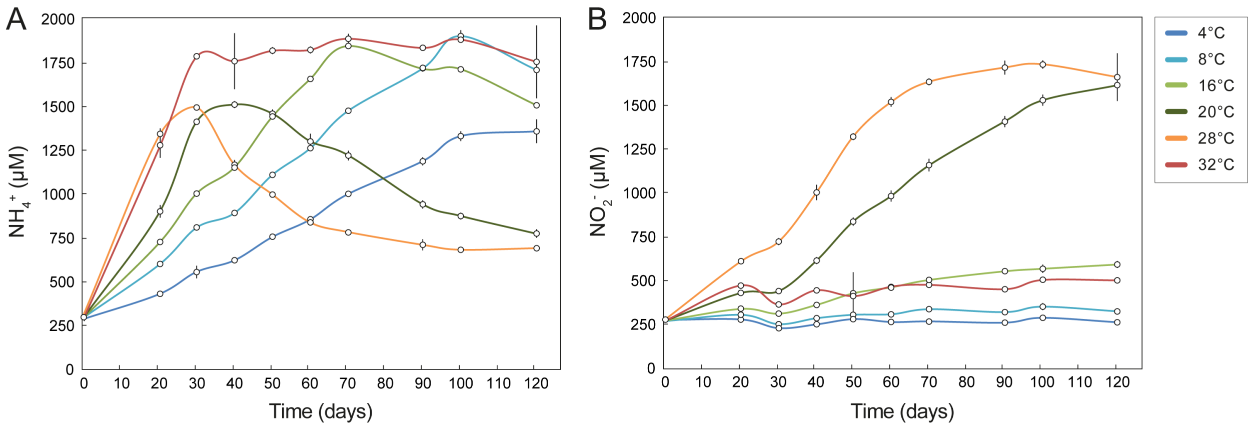

Figure S1. Net ammonia oxidation dynamics in enrichment cultures of *Ca.* N. arcticus Kfb over a temperature range from 4°C to 32°C. Cultures were grown in mineral medium supplemented with 1 mM urea. **(A)** Cumulative net NH_4^+ consumption, and **(B)** cumulative net NO_2^- production. Lines with different colors represent cultures at different temperatures, according to the legend in the figure. Error bars represent the standard deviation of triplicate cultures; some error bars are smaller than the symbols and thus are not visible.

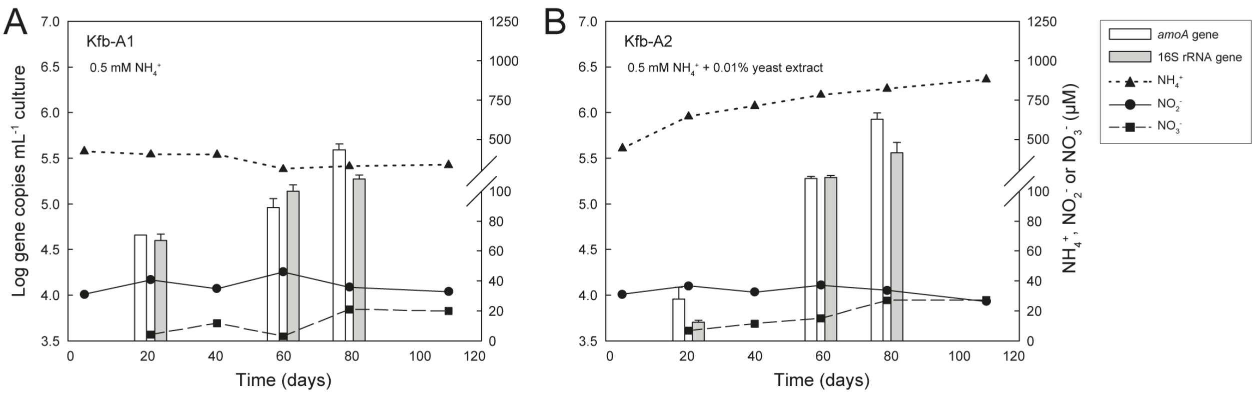

Figure S2. **Growth of AOA in two early enrichment cultures at 4°C.** Cultures were supplemented with either **(A)** 0.5 mM NH_4^+, or **(B)** 0.5 mM NH_4^+ and 0.01% yeast extract. Growth was determined based on quantification of archaeal *amoA* (white bars) and thaumarchaeal 16S rRNA (gray bars) genes with qPCR. Error bars represent the standard deviation of triplicate measurements; some error bars are smaller than the symbols and thus are not visible. For more details see text.

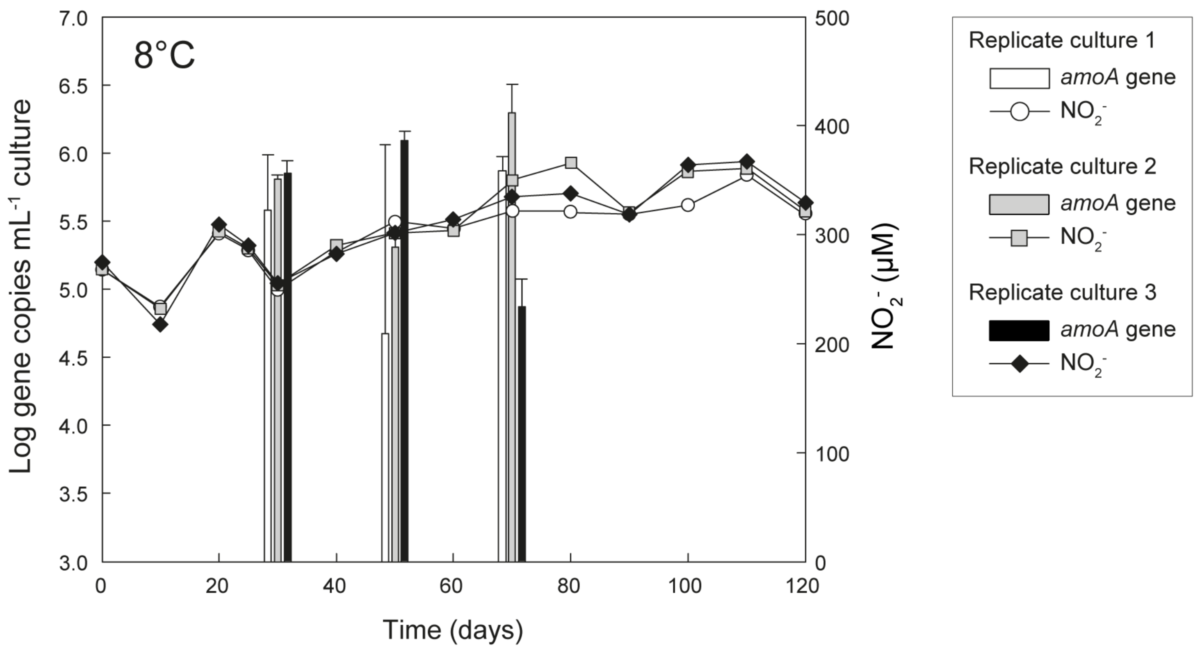

Figure S3. Growth and ammonia oxidation by *Ca.* N. arcticus Kfb in three individual cultures at 8°C. Cultures were grown in mineral medium supplemented with 1 mM urea, and incubated in parallel with several cultures derived from the same inoculum, but incubated at different temperatures (Figure 2). Growth was determined based on quantification of archaeal *amoA* genes (bars) with qPCR; error bars represent the standard deviation of triplicate measurements. Solid lines represent cumulative net NO_2^- production. White, gray and black bars and symbols represent three different replicate cultures, respectively.

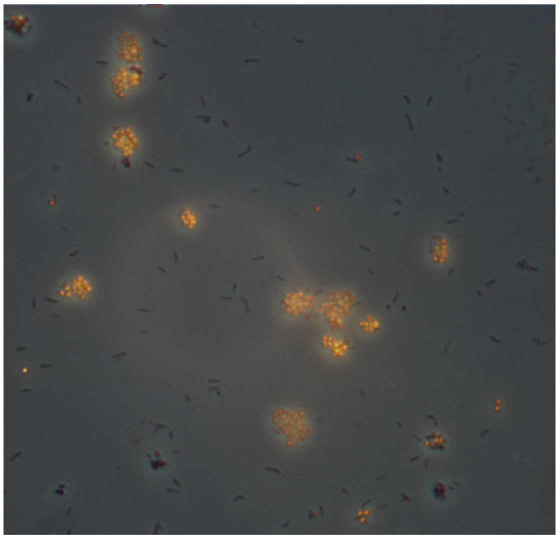

Figure S4. Micrographs illustrating the enrichment state of *Ca*. N. arcticus Kfb. Combined phase contrast and epifluorescence (DOPE-FISH with archaea-specific probe ARCH915) micrographs.

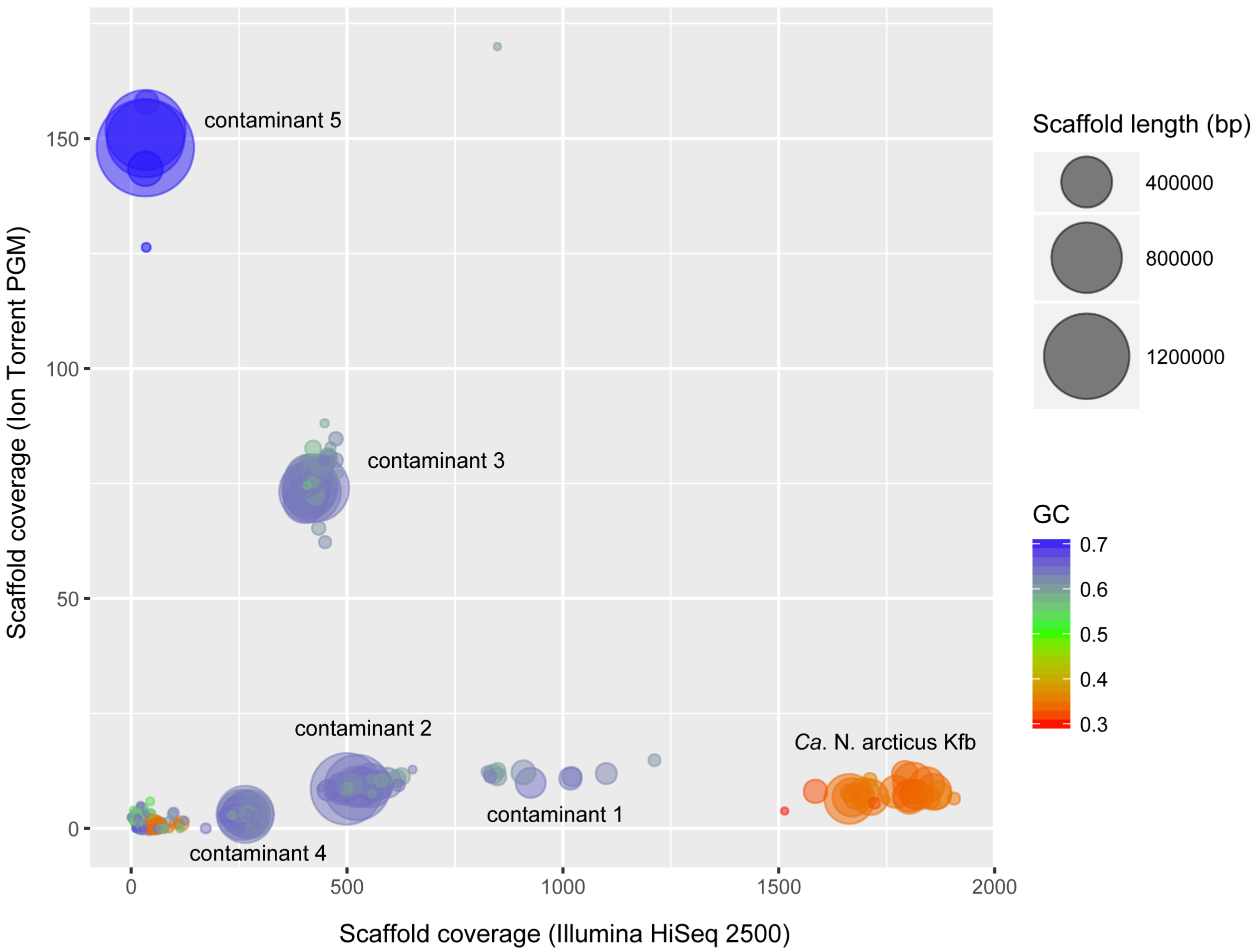

Figure S5. Differential coverage binning of metagenome scaffolds from *Ca.* N. arcticus Kfb enrichment cultures. The binning used the differential coverage of Illumina (forward and reverse reads) and Ion Torrent PGM sequencing runs, and is thus independent of sequence composition. Each circle represents a scaffold, colored by GC (%) content and scaled by the square root of their length. Only scaffolds from the metagenome assembly >2 kbp and an Illumina scaffold coverage <2000 are shown. Clusters of scaffolds with similar colors represent potential genome bins. The orange cluster was assigned to *Ca.* N. arcticus Kfb, whereas the blue colored clusters are assigned to bacterial contaminants in the enrichment cultures. For better visualization, one scaffold of *Ca.* N. arcticus Kfb containing 16S and 23S rRNA genes, with Illumina coverage 4894 and Ion Torrent coverage 142, is not shown in the plot.

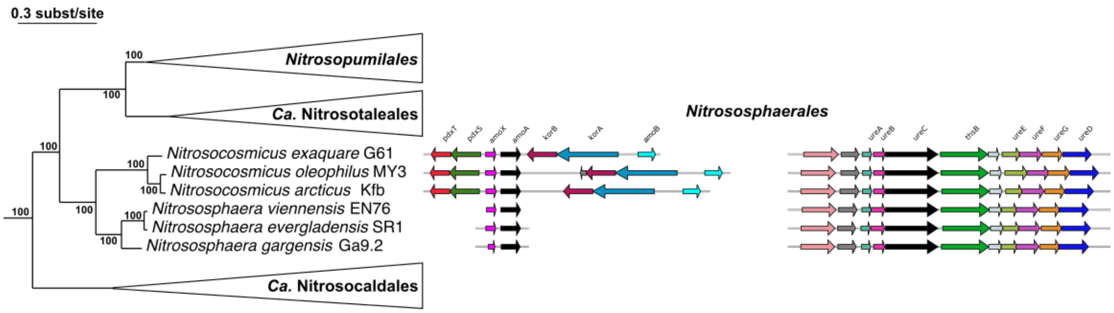

Figure S6. Phylogenetic tree of class *Nitrososphaeria* and genomic loci encoding ammonia monooxygenase (AMO) and urease in genomes of the order *Nitrososphaerales*. The tree was inferred by maximum likelihood based on a concatenated alignment of 59 ribosomal proteins (Abby et al. 2018). In *Ca.* Nitrosocosmicus genomes, *amoC* copies are dispersed between different loci (2, 3 and 2 copies for *Ca.* N. arcticus Kfb, *Ca.* N. oleophilus MY3 and *Ca.* N. exaquare G61, respectively), whereas in *Nitrososphaera* genomes, both *amoB* (one copy per genome) and *amoC* (several copies per genome) are located at other genomic locations (see Figure 4 in (Abby et al. 2018). Genetic loci representations were drawn using GeneSpy (Garcia et al. 2019).

References

Abby, S. S., M. Melcher, M. Kerou, M. Krupovic, M. Stieglmeier, C. Rossel, K. Pfeifer, and C. Schleper. 2018. 'Candidatus Nitrosocaldus cavascurensis, an Ammonia Oxidizing, Extremely Thermophilic Archaeon with a Highly Mobile Genome', *Frontiers in Microbiology*, 9: 28.

Alves, R. J. E., B. Q. Minh, T. Urich, A. von Haeseler, and C. Schleper. 2018. 'Unifying the global phylogeny and environmental distribution of ammonia-oxidising archaea based on amoA genes', *Nat Commun*, 9: 1517.

Alves, R. J., W. Wanek, A. Zappe, A. Richter, M. M. Svenning, C. Schleper, and T. Urich. 2013. 'Nitrification rates in Arctic soils are associated with functionally distinct populations of ammonia-oxidizing archaea', *ISME J*, 7: 1620-31.

Garcia, P. S., F. Jauffrit, C. Grangeasse, and C. Brochier-Armanet. 2019. 'GeneSpy, a user-friendly and flexible genomic context visualizer', *Bioinformatics*, 35: 329-31.
